# PrC-210 Protects against Radiation-Induced Hematopoietic and Intestinal Injury in Mice and Reduces Oxidative Stress

**DOI:** 10.3390/antiox12071417

**Published:** 2023-07-13

**Authors:** Vidya P. Kumar, Shukla Biswas, Gregory P. Holmes-Hampton, Torsten Goesch, William Fahl, Sanchita P. Ghosh

**Affiliations:** 1 Armed Forces Radiobiology Research Institute, Uniformed Services University of the Health Sciences, Bethesda, MD 20889, USA; vidya.kumar.ctr@usuhs.edu (V.P.K.); gregory.holmes-hampton@usuhs.edu (G.P.H.-H.); 2 Obvia Pharmaceuticals Ltd., Madison, WI 53705, USA; torsten@goesch.com (T.G.); fahl.bill@gmail.com (W.F.); 3 Wisconsin Institutes for Medical Research, Department of Oncology, University of Wisconsin-Madison, Madison, WI 53705, USA

**Keywords:** acute radiation syndrome, antioxidant, hematopoietic radiation injury, PrC-210, prophylactic countermeasure

## Abstract

The development of safe, orally available, and effective prophylactic countermeasures to protect our warfighters is an unmet need because there is no such FDA-approved countermeasure available for use. Th 1-Propanethiol, 3-(methylamino)-2-((methylamino)methyl) (PrC-210), a synthetic small molecule, is a member of a new family of aminothiols designed to reduce toxicity while scavenging reactive oxygen species (ROS). Our study investigated the protective role of a single oral administration of PrC-210 against radiation-induced hematopoietic and intestinal injury in mice. Pre-treatment with PrC-210 significantly improved the survival of mice exposed to a lethal dose of radiation. Our findings indicated that the radioprotective properties of PrC-210 are achieved by accelerating the recovery of the hematopoietic system, stimulating bone marrow progenitor cells, and ameliorating additional biomarkers of hematopoietic injury. PrC-210 pre-treatment reduced intestinal injury in mice exposed to a lethal dose of radiation by restoring jejunal crypts and villi, reducing translocation of bacteria to the spleen, maintaining citrulline levels, and reducing the sepsis marker serum amyloid A (SAA) in serum. Finally, PrC-210 pre-treatment led to a significant reduction (~10 fold) of Nos2 expression (inducible nitric oxide) in the spleen and decreased oxidative stress by enhancing the antioxidant defense system. These data support the further development of PrC-210 to receive approval from the FDA to protect warfighters and first responders from exposure to the harmful effects of ionizing radiation.

## 1. Introduction

Ionizing irradiation induces the sequelae of cellular, tissue, and organ injuries, which ultimately lead to total body injury and lethality depending on the absorbed dose [1,2,3,4]. Following radiation exposure, free radicals, including superoxide and a hydroxyl radical, are generated by the hydrolysis of water in cells within a fraction of a second [3,5,6,7]. The Walter Reed Army Institute of Research screened more than 4400 agents to identify drugs that protect against injuries caused by the ionizing radiation from nuclear/radiological detonations. Amifostine (WR-2721, 2-[(3-aminopropyl) amino] ethanethiol dihydrogen phosphate) was initially developed during the Cold War by the Walter Reed Army Institute of Research as a radioprotectant because of its ability to scavenge the free radicals generated by radiation exposure [8]. The authors [8] reported that WR-2721 is (i) currently approved by the Food and Drug Administration (FDA) for human use for severe xerostomia (dry mouth and the associated pathologic sequelae of the oral cavity) in patients with head and neck cancers undergoing intense local radiotherapy; (ii) found to be inherently toxic when administered at high, cytoprotective doses; (iii) hypotensive in nature, producing both upper and lower gastrointestinal disturbances (nausea/vomiting can yield adverse behavioral responses and decremented performances); and (iv) not effective when topically applied to human skin. These WR-2721 qualities lead to adverse behavioral responses and decreased performance [8,9]. As a result, this promising radioprotector was not considered a viable option for the radioprotection of either first responders or civilian populations. Investigators tried, through various approaches (dosing regimens, modulating the drug delivery, and modifying the drug itself), to limit its side effects while retaining its robust radioprotective properties [9,10,11]. Such efforts led to the invention of a new aminothiol, 1-propanethiol, 3-(methylamino)-2-((methylamino)methyl) (PrC-210), through a three-year “directed evolution” discovery process [10]. PrC-210 was found to be (i) about 10% more effective than amifostine, based upon its Dose Reduction Factor (DRF), when systemically administered; (ii) orally active; (iii) free of limiting amifostine side effects, i.e., it does not induce vomiting or hypotension in pre-clinical ferret and rodent models; (iv) preventive of Grade 2–3 radiodermatitis when topically applied as well as when applied by intraperitoneal (IP) administration before skin irradiation; and (v) protective of mice from a lethal dose of whole-body radiation (8.75 and 9.0 Gy), following prophylactic IP administration [9,11].

PrC-210 also functions as a post-exposure agent to mitigate the development of Acute Radiation Syndrome (ARS) following radiation exposure in both ICR (CD-1) and C57BL/6 male mice [12]. PrC-210, when administered once by the IP route 24 h post-irradiation, significantly enhanced survival in both strains of mice exposed to a lethal dose of radiation [12]. PrC-210-treated mice demonstrated a significant increase in body weight post-exposure, with accelerated bone marrow recovery compared to non-treated animals [11]. MRI imaging of live mice showed protection of the intestinal structure and restoration of the jejunal villi and surface epithelium [12].

In an effort to identify promising prophylactic countermeasures to be used by military personnel before deployment for rescue or cleanup operations following a radiation/nuclear event, we demonstrated the efficacy of a single dose of orally administered PrC-210 in CD2F1 male mice. We used this strain because we developed this model and extensively used it to test other medical countermeasures for acute radiation syndrome (ARS) [13,14,15]. In this study, we showed that PrC-210, administered 1 h pre-total-body irradiation (pre-TBI), accelerated recovery from radiation-induced peripheral blood cytopenia and restored sternal bone marrow, protected bone marrow progenitor cells, and attenuated protein biomarkers of H-ARS (hematopoietic acute radiation syndrome). In addition, we reported that PrC-210 pre-administration protected the jejunal crypt and inflammatory markers to ameliorate gastrointestinal injury in animals exposed to a lethal dose of radiation.

## 2. Materials and Methods

### 2.1. Animals and Veterinary Care

For the studies reported here, 12–14-week-old male CD2F1 mice were purchased from Envigo (Indianapolis, IN, USA). The mice were housed in the Armed Forces Radiobiology Research Institute’s (AFRRI) vivarium, accredited by the Association for Assessment and Accreditation of Laboratory Animal Care International. The animals were provided the Harlan Teklad Rodent Diet 8604 (Envigo) and acidified water (pH 2.5–3.0) ad libitum, were housed under an automated lighting system providing a 12 h light/dark cycle, and were acclimatized for at least five days before the start of each study [13]. Veterinary care was available throughout the course of the study, and animals were examined by the research and veterinary staff as warranted for clinical signs or changes in appearance [14].

### 2.2. Ethics Statement

All animal procedures were reviewed and approved by the AFRRI Institutional Animal Care and Use Committee (IACUC) using principles outlined in the National Research Council’s Guide for the Care and Use of Laboratory Animals. Mice were considered moribund when they showed an inability to remain upright, cold, unresponsive, or displayed decreased or labored respiration. Moribund mice were euthanized humanely according to American Veterinary Medical Association (AVMA) guidelines, including CO_2_ inhalation and confirmatory cervical dislocation [15].

### 2.3. PrC-210 Administration

PrC-210 HCl (MW: 220) was synthesized by Fahl et al. [16]. PrC-210 was supplied to AFRRI in a powder form and was formulated in sterile water before use and protected from light. Either drug or water (100 µL) (control) was administered orally as a single dose 60 min pre-TBI exposure at indicated concentrations.

### 2.4. Radiation

A single bi-lateral exposure of 60Co gamma at an estimated dose rate of 0.6 Gy/min at three radiation doses (7, 9.35, and 9.75 Gy) in the AFRRI radiation facility was used for total-body irradiation (TBI) where the mice were placed in ventilated Lucite™ boxes arranged in an array using plastic racks during the exposure. Dosimetry was carried out with the alanine electron spin resonance (ESR) dosimetry system (ASTM 1996) as published earlier [14]. The dose rates to water in cores of acrylic mouse phantoms were measured as described earlier [14]. The uniformity of the radiation field was within ±2%.

### 2.5. Acute Toxicity Study

Prior to survival studies on irradiated mice, a basic safety study was performed by administering PrC-210 to male CD2F1 mice at a 900 mg/kg dose via oral gavage (PO). Animals were divided into two groups, with and without fasting. Fasted animals were deprived of feed for 5 h before drug administration by removing food from those cages. Food was replaced once the drug had been administered. Mice were administered either PO PrC-210 or water as the vehicle control. Age-matched naive mice were used as controls. All mice were monitored for acute (1 to 4 h) signs of toxicity after PO administration, then daily for 14 d. Animals were monitored for signs of acute toxicity, including decreased activity, squinting eyes, hunching, labored breathing, or mortality. The weights of the animals were recorded at various intervals during the study. Small amounts of blood (20 µL) were collected for complete blood count (CBC) via a submandibular vein in Minivette POCT (Sarstedt, Germany) on selected days when body weights were recorded. CBC measurements were made on a Heska HT5 hematoanalyzer (Heska Inc., Loveland, CO, USA). All animals were euthanized on day 14, and a gross necropsy was conducted to identify any abnormal pathology in major organs. Blood was collected by cardiocentesis for serum chemistry analyses of hepatic and renal panels at the end of the study.

### 2.6. Thirty-Day Survival Efficacy and PrC-210 Dose-Response Studies

The initial study to determine survival efficacy consisted of testing one drug dose (450 mg/kg) of PrC-210, one route of administration (PO), and one radiation dose (9.35 Gy). This radiation dose was used because it produces ~70% lethality in this strain of mice [13]. CD2F1 male mice were weighed, and animals outside ±10% of the mean weight were excluded and randomized into groups. PrC-210 was administered orally (PO) once, one hour prior to irradiation. There were two groups, “Fasting” and “Fed,” for the drug- and water-treated groups. Feed was removed from the “Fasting” groups 5 h prior to drug administration. There were 24 animals (identified by tail tattoo) per dietary condition group for PrC-210 and its vehicle, water. All animals received radiation at an estimated 0.6 Gy per/min dose rate in the AFRRI 60Co gamma radiation facility. Animals were monitored daily for radiation-induced clinical symptoms (minimum three times a day during the period of peak mortality) for 30 days and euthanized at the completion of the observational period. Survival efficacy was plotted as Kaplan–Meier plots.

In the dose optimization studies, four doses of PrC-210 (250, 450, 600, and 800 mg/kg by PO) and water as control were tested. PrC-210 was administered PO once, one hour prior to irradiation in CD2F1 male mice (n = 24/group). Feed was not removed for any of the animals in this study. They were monitored daily (minimum three times a day during the period of peak mortality) for 30 days and euthanized at the completion of the observational period.

### 2.7. Assessment of Hematological Recovery by PrC-210 Pretreatment

#### 2.7.1. Effect of PrC-210 on Peripheral Blood Cell Recovery Post-TBI

The animals received either 0 or 7 Gy radiation at a dose rate of 0.6 Gy/min TBI. A non-lethal dose of 7 Gy was chosen to monitor hematopoietic recovery over 30 days [14]. All animals were treated with water (vehicle) or PrC-210 (450 mg/kg), PO at 1 h prior to TBI (n = 10/group). Submandibular blood collection was carried out for eight time points, at 2 h and days 1, 3, 7, 10, 14, 21, and 30 post-TBI. Animals were euthanized 30 days after irradiation. About 20 µL of blood was collected from the submandibular vein in EDTA-coated tubes following a standard protocol, and CBC/differential analysis was performed. All animals were monitored over 30 days for recovery following radiation exposure.

#### 2.7.2. Effect of PrC-210 on the Bone Marrow Cellularity Post-TBI

All animals were administered either water or PrC-210 (450 mg/kg), PO 1 h prior to TBI (n = 10/group). Tissues were collected from animals irradiated at 0 Gy and 7 Gy TBI at various time points (2 h, days 1, 3, 7, 10, 14, and 30 post-TBI). A clonogenic assay was performed with cells harvested from femoral bone marrow, as described previously [14]. Sternal histopathology was carried out on the sterna fixed in 10% neutral-buffered formalin, as described previously [17]. The bone marrow cellularity was qualitatively evaluated within the sternebrae, and megakaryocytes were counted by averaging the number of cells per 10 (40× high-powered fields (HPFs).

#### 2.7.3. Effect of PrC-210 on the Biomarkers of Bone Marrow Aplasia

Mouse erythropoietin (EPO) and Flt3 ligand (Flt3L) are established biomarkers of radiation-induced bone marrow damage [14,18]. Sandwich mouse ELISA kits were purchased from R&D Systems Inc. (Minneapolis, MN, USA). The cytokine detection limits were >20 pg/mL and >5 pg/mL for EPO and Flt3L, respectively. The quantitative levels of EPO, Flt3L, and SAA were evaluated from serum samples collected on days 0 (2 h), 1, 3, 7, 10, 14, and 30 post-TBI (7 Gy) following standard protocols from the vendor. Represented data are mean ± SEM for n = 6 mice.

### 2.8. Total RNA Extraction from Spleen and Oxidative Stress Pathway Analysis

Spleens harvested from 7 Gy irradiated and naive mice were immediately snap frozen in liquid nitrogen and stored at −80 °C. The frozen tissue was homogenized by brief sonication on ice, and total RNA was extracted using a mirVana total RNA isolation kit (Life Technologies, Frederick, MD, USA; #AM 1560) following the protocol of the manufacturer [19]. RNA yield and quality were analyzed on a NanoDrop spectrophotometer ND-1000 (ThermoFisher Scientific Inc., Rockville, MD, USA). cDNA was synthesized from the RNA using an RT2 First Strand kit (Qiagen, Germantown, MD, USA). RT^2^ Profiler™ PCR Array Mouse Oxidative Stress and Antioxidant Defense (PAMM-065ZA) RT PCR array were performed using a QuantStudio 3 PCR Machine, and the analysis was completed using the Qiagen GeneGlobe Data Analysis Center online [19].

### 2.9. Assessment of Gastrointestinal Recovery by Pre-Treatment of PrC-210

All animals were administered either water or PrC-210 (450 mg/kg), PO 1 h prior to 9.75 Gy TBI (n = 10/group). An approximately 90% lethal dose of 9.75 Gy was chosen in this study to assess gastrointestinal damage in this strain and the effect of PrC-210. Blood and tissues were collected at various time points (2 h and days 1, 3, 7, and 9 post-TBI). The jejunum, liver, and spleen were flash-frozen in liquid nitrogen upon collection and stored at −80 °C until used for bacterial translocation studies [20]. For histopathological evaluation, a portion of jejunum was fixed in 10% neutral buffered formalin for 24 h and sectioned and stained with hematoxylin and eosin (H&E). The serum was separated and stored at −80 °C until used for ELISA analysis [20].

#### 2.9.1. Intestinal Crypt Colony Assay

A microcolony crypt survival assay was performed as described previously [20,21,22]. Segments of proximal jejunum were obtained at all time points (days 1, 3, 7, and 9 post-TBI), fixed, and embedded so that four transverse sections were obtained per specimen, cut at 3–5 μm, and stained with hematoxylin and eosin (H&E). Damage to the intestinal structure was evaluated by assessing the shortening of villi in the stained sections. Crypt viability was determined on days 1, 3, and 7 post-TBI by the presence of at least 10 epithelial cells, a lumen, and a minimum of one Paneth cell. Counting the number of surviving crypts in each circumference was blinded by two individuals in order to reduce the subjectivity, and five circumferences were counted from each mouse. Crypt survival was expressed as the average number of surviving crypts from two sections per mouse and six mice/group. The average from each mouse was considered as a single value for statistics, and representative images were shown.

#### 2.9.2. Estimation of Bacterial DNA in Spleen and Liver Following Radiation

Bacterial load in the liver and spleen due to translocation from the damaged gut was determined and quantified by real-time PCR using the 16S rRNA gene consensus sequence, which is highly conserved in bacteria [20]. DNA was extracted from previously frozen tissues (jejunum, liver, spleen) from irradiated (9.75 Gy TBI) mice pre-treated with either water or PrC-210 by first cutting the tissue (roughly 25–30 mg) into small pieces and placing in a 1.5 mL microcentrifuge tube. Next, proteinase K was added to the sample and incubated at 56 °C until the tissue was lysed. The DNA was prepared using the Qiagen DNeasy mini kit. Following the DNA isolation, the yield was quantified using a nanodrop spectrophotometer. Using fresh aseptically prepared aliquots of SYBR Green Master Mix and PCR water, samples were prepared for PCR with primers for the 16S rRNA to quantify bacterial load [20].

#### 2.9.3. Effect of PrC-210 on the Serum Citrulline and SAA 

Citrulline is a known biomarker of intestinal damage [20]. Citrulline levels were quantified using a citrulline assay kit (Cell Biolabs, San Diego, CA, USA), in accordance with the instructions of the manufacturer, for animals exposed to 9.75 Gy TBI. Serum Amyloid (SAA) is a reported sepsis biomarker following radiation injury [18]. Sandwich mouse ELISA kits were purchased from R&D Systems Inc. (Minneapolis, MN, USA). The quantitative levels of SAA were evaluated from serum samples collected on days 1, 3, and 7 post-TBI (9.75 Gy) in accordance with the instructions of the manufacturer. Represented data are mean ± SEM for n = 6 mice.

### 2.10. Statistical Analysis

Survival data were plotted as Kaplan–Meir plots. A log-rank test was used to evaluate the survival curves for significant alterations, and a Fisher’s exact test was used to evaluate differences in the surviving proportions at the conclusion of the study [14]. Analysis of variance (ANOVA) and *t*-test were used to determine if there was a significant difference among the groups [14]. Data are reported as means and standard errors.

## 3. Results

### 3.1. PrC-210 Was Found to Be Safe Administered Orally

In a 14-day acute toxicity study, PrC-210 was found to be safe in CD2F1 male mice when administered once orally at 900 mg/kg body weight (0.5 Maximum Tolerated Oral Dose) 60 min prior to radiation. The animals, both fasted and fed groups, showed no clinical symptoms of toxicity and gained weight during the study in a similar manner as the naive group (Supplementary Figure S1A). No abnormal pathology was found in any of the major organs during gross necropsy. CBC analysis for white blood cells (WBC), neutrophils (NEU), platelets (PLT), and lymphocytes (LYMP), and serum chemistry analysis of hepatic and renal panels (BUN Creatinine, AST, Total Protein, ALKP, ALT) in both fasted and fed groups were found to be in the normal range at the end of the study (day 14 after PrC-210 administration) indicating no PrC-210 toxicity at the dose administered to the animals (Supplemental Figure S1B,C).

### 3.2. Survival of Lethally Irradiated Mice from a Single Dose of Pre-Administration of PrC-210

**Figure 1:**
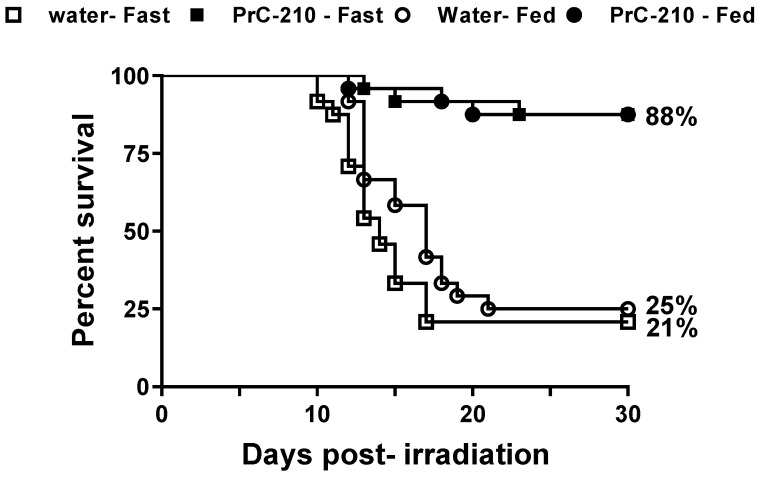
Survival of animals administered PrC-210 (450 mg/kg) or water PO 1 h prior to exposure to 9.35 Gy TBI. Animals administered PrC-210 included two cohorts (n *=* 24/cohort); (i) fasted for 5 h prior to drug administration, (■) and (ii) fed ad libitum (●); survival for both groups was 88%. Percent survival for animals administered the vehicle (included two cohorts (n *=* 24/cohort)) was 21% in the fasted group (□) and 25% in the fed ad libitum group (○). The log-rank analysis of the water and PrC-210 survival curves had a p-value of <0.0001 and Fisher’s exact test with a *p* < 0.0001 for both conditions. There were no significant differences between the fasted and fed animals.

Thirty-day survival efficacy in CD2F1 mice following TBI was tested at a dose of 450 mg/kg (0.25 Maximum Tolerated Oral Dose) in mice administered 1 h prior to exposure to 9.35 Gy at the estimated rate of 0.6 Gy/min. The drug-treated cohorts had 88% survival in both the fasted and fed groups compared to 21% survival in control fasted and 25% in control fed groups (Figure 1, n *=* 24/group). This significant increase in survival with PrC-210 treatment indicates that the free radical scavenger PrC-210 can be developed further as a radioprotectant. The log-rank test indicated a highly significant difference (*p* < 0.0001) when the PrC-210 survival curves were compared to their respective vehicle-treated groups (i.e., fasted or fed). A Fisher’s exact test of surviving proportions between the PrC-210-treated and their corresponding controls also showed highly significant differences (*p* < 0.0001), indicating that survival was significantly enhanced with PrC-210 treatment compared to the control. Neither log-rank nor Fisher’s exact test were significant when comparing the fasted and fed PrC-210 or the fasted and fed vehicle groups.

### 3.3. PrC-210 Dose Response in 30-Day Survival Studies

**Figure 2:**
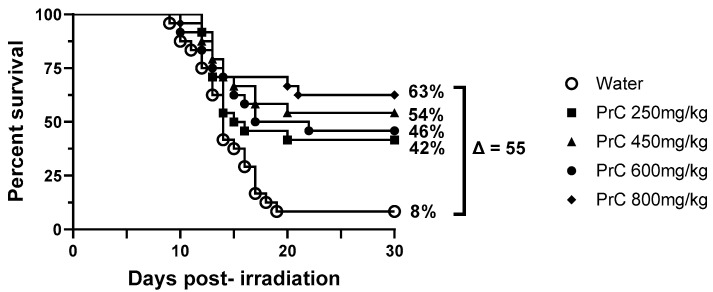
Dose optimization of PrC-210. Groups included vehicle (○) or PrC-210 at doses of 250 mg/kg (■), 450 mg/kg (▲), 600 mg/kg (●), and 800 mg/kg (♦). Survival for the 800 mg/kg groups was 63%, the 450 mg/kg group was 54%, the 600 mg/kg group was 46%, the 250 mg/kg was 42%, and the survival in the group administered water was 8%. Log-Rank analyses of all PrC-210 groups compared to water were significant (*p* = 0.0001–*p* = 0.0179), and Fisher’s exact test of all PrC-210 groups compared to water was as significant (*p* = 0.0002–*p* = 0.0173). There were no significant differences between groups administered PrC-210.

In order to determine the optimum dose of PrC-210 administered 1 h pre-TBI, a range of drug doses (250 to 800 mg/kg) were tested in mice (n *=* 24) irradiated at 9.35 Gy (Figure 2). Since there was no difference in 30-day survival efficacy in the fasted and fed group (Figure 1), we excluded the fasted groups in the subsequent studies reported here. Survival of the groups was 63% for animals administered 800 mg/kg PrC-210, 54% for animals administered 450 mg/kg PrC-210, 46% for animals administered 600 mg/kg PrC-210, and 42% for animals administered 250 mg/kg PrC-210; these were all compared to 8% survival for animals administered the vehicle (water). All groups administered PrC-210 showed significant differences in the survival curves (Log-Rank; *p =* 0.0001, *p =* 0.0054, *p =* 0.0007, and *p =* 0.0179 for 800 mg/kg, 450 mg/kg, 600 mg/kg, and 250 mg/kg vs. water, respectively). Additionally, survival proportions for all groups administered PrC-210 were significantly increased compared to those administered water (Fisher’s exact test *=* 0.0002, *p =* 0.0078, *p =* 0.0013, and *p =* 0.0173 for 800 mg/kg, 450 mg/kg, 600 mg/kg, and 250 mg/kg *vs.* water, respectively). There were no significant differences (Log-Rank or Fisher’s exact test) between the survival curves or surviving proportions for any of the groups administered PrC-210 compared to any other dose. Despite this, the difference in survival for the 450 mg/kg (54%) dose was higher than the dose below (250 mg/kg, 42%) and above (600 mg/kg, 46%). Therefore, 450 mg/kg was selected to develop PrC-210 further to test various endpoints for hematopoietic and gastrointestinal injury following non-lethal and lethal doses of ionizing radiation.

### 3.4. CBC Recovery

**Figure 3:**
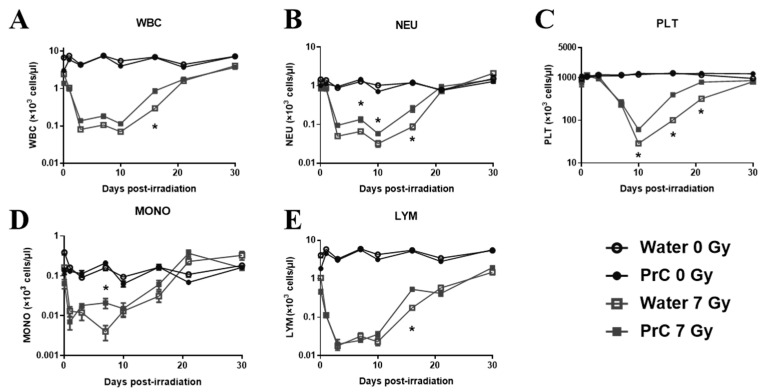
Recovery of peripheral blood cells following radiation injury. (**A**). white blood cells (WBCs), (**B**). neutrophils (NEU), (**C**). platelets (PLT), (**D**). monocytes (MONO), and (**E**). lymphocytes (LYM)). Day 0 represents 8 h post-irradiation. Non-irradiated mice treated with water (○) and PrC-210 (●) and irradiated (7 Gy) mice treated with water (□) and PrC-210 (■). Data shown are mean ± standard error of the mean (SEM) for n *=* 10 mice. Significant differences (*p* < 0.001–0.0125) between PrC-210-treated and water-treated irradiated groups by ANOVA are indicated with an asterisk (*). Some data points in the figure do not have error bars that are visible because they are smaller than symbols.

Hematological recovery was evaluated by comparing peripheral blood cell counts (white blood cells (WBC, Figure 3A), neutrophils (NEU, Figure 3B), platelets (PLT, Figure 3C), monocytes (MON, Figure 3D) and lymphocytes (LYM, Figure 3E) of irradiated mice treated with or without PrC-210 administered pre-TBI. CBC was monitored at each time (2 h and days 1, 3, 7, 10, 14, 21, and 30 days following radiation) from the same cohort of mice (n *=* 10) from the four groups (two non-irradiated and two irradiated, water and PrC-210). A non-lethal dose of 7 Gy was used to induce hematopoietic injury and monitor its recovery through the study period (30 days) following exposure so that no animals had to be euthanized before completion. There was no significant effect of PrC-210 on non-irradiated mice when compared with the water-treated group over the testing period (Figure 3). While monitoring the recovery profile of the two irradiated groups following irradiation, we observed significantly accelerated recovery with the PrC-210 pre-treated group compared to its control (*p =* 0.00026) on day 14 for WBC; days 7, 10 (*p =* 0.037), and 14 (*p =* 0.0005) for NEU; days 10, 14, and 21 for PLT (*p* < 0.0001 on all days); day 7 for MONO (*p =* 0.05); and day 14 for LYM (*p* < 0.0001). These data indicate that PrC-210 pre-treatment accelerated recovery of peripheral blood cytopenia in animals exposed to ionizing radiation, manifesting H-ARS.

### 3.5. Hematopoietic Progenitor Cells Recovery

**Figure 4:**
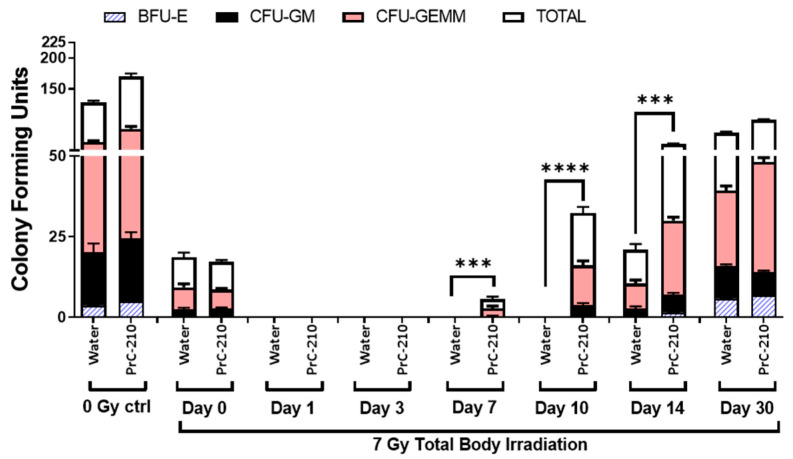
Recovery of femoral bone marrow by clonogenic assay. Bone marrow colony-forming units (CFU) were estimated in the femurs of the animals administered either water or PrC-210 (450 mg/kg) 1 h prior to irradiation (7 Gy) and non-irradiated animals (0 Gy) to quantify the recovery. Significant difference *** *p* ≤ 0.001, **** *p* ≤ 0.0001.

Hematopoietic progenitor cells are sensitive to radiation because they are vital due to their self-renewal and differentiation ability [23]. In addition to detrimental effects on peripheral blood cells, irradiation also negatively affects hematopoietic progenitor cells [23]. Clonogenic assays were carried out to evaluate the extent of damage caused by irradiation and possible recovery by PrC-210 treatment administered 24 h pre-TBI. Colony-forming unit (CFU) assays measured CFU-GM, CFU-GEMM, CFU-E, and BFU-E to evaluate the function of hematopoietic cells. As shown in Figure 4, no colonies were observed up to day 10 post-TBI at 7 Gy in the vehicle-treated group. However, colonies started growing on day seven post-TBI in the PrC-210 treated group, and numbers were significantly higher (*p* < 0.0001) on day 14 in the PrC-210 group compared to the control. Accelerated regeneration of bone marrow progenitor cells in the PrC-210 treated group indicated that the cellular functions affected by irradiation and hematopoietic injury that may cause lethality can be restored by PrC-210 pre-treatment.

### 3.6. PrC-210 Pre-Treatment Restored Overall Cellularity and Protected Megakaryocytes

**Figure 5:**
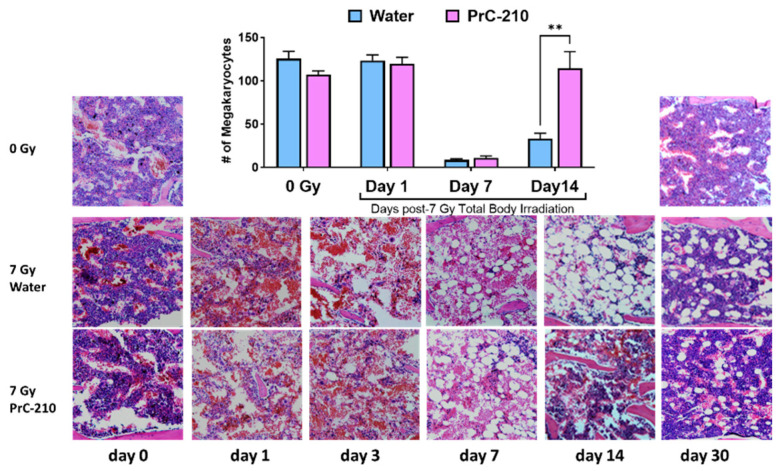
Recovery of cellularity of sternal bone marrow was determined on days 0 (8 h post-TBI), 1, 3, 7, 14, and 30 post-TBI (7 Gy). The number of megakaryocytes was counted in all four groups (water-treated (0 and 7 Gy) and PrC-210-treated (0 and 7 Gy). The irradiated PrC-210 group showed significant recovery (** *p* < 0.01) compared to the saline group. Representative H&E-stained sections of sternal bone marrow are shown. Higher loss of cellularity and higher numbers of adipocytes are seen in the irradiated water-treated group compared to the PrC-210-treated group.

Histopathological analysis was carried out on sternal bone marrow to evaluate the effect of PrC-210 on overall cellularity, loss of myeloid and erythroid cells, and megakaryocytes. As expected, radiation exposure induced a substantial decrease in bone marrow cellularity, including the number of megakaryocytes, replacing the bone marrow cells with adipocytes. Sternal bone marrow from irradiated groups was compared with non-irradiated groups throughout the study. The high magnification image (Figure 5) clearly shows a progression of severe hematopoietic depletion over time from days 1 to 14 in the irradiated control group with loss of cellularity and filling of the marrow cavity with adipocytes. The PrC-210 pre-treated mice showed slow recovery over time to moderate cellularity levels and restored megakaryocytes on day 14, which was significantly higher than the control group (Figure 5).

### 3.7. PrC-210 Attenuates Radiation-Induced Induction of EPO and Flt3L

**Figure 6:**
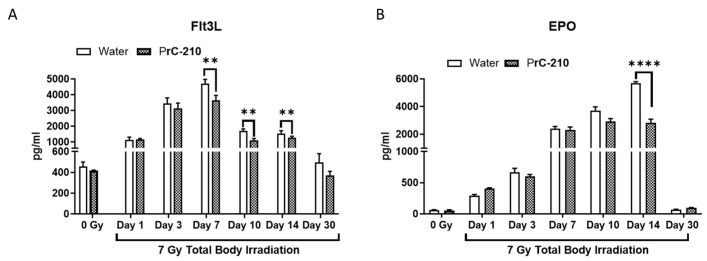
Serum levels of FLT3-L (**A**) and EPO (**B**) on various days post-TBI (7 Gy) were measured by ELISA. Significantly lower (** *p* ≤ 0.01) FLT3L levels were observed from day seven onwards in the PrC-210-treated group. In the case of EPO levels, significant recovery (**** *p* ≤ 0.0001) by PrC-210 treatment was seen on day 14 post-TBI when compared to the water group. Represented data are mean ± standard error of the mean (SEM) for n *=* 4 mice.

To confirm the effect of PrC-210 on radiation-induced hematopoietic recovery, we measured the circulatory markers EPO and Flt3L. As expected, both markers increased after radiation with time [18]; however, they returned to the basal level by day 30. Pre-administration of PrC-210 significantly reduced Flt3L on days 7 to 14 and EPO on only day 14 post-exposure (Figure 6A,B).

### 3.8. PrC-210-Mediated Hematopoietic Recovery Was Correlated with Upregulation of Nitric Oxide Synthase 2

**Figure 7:**
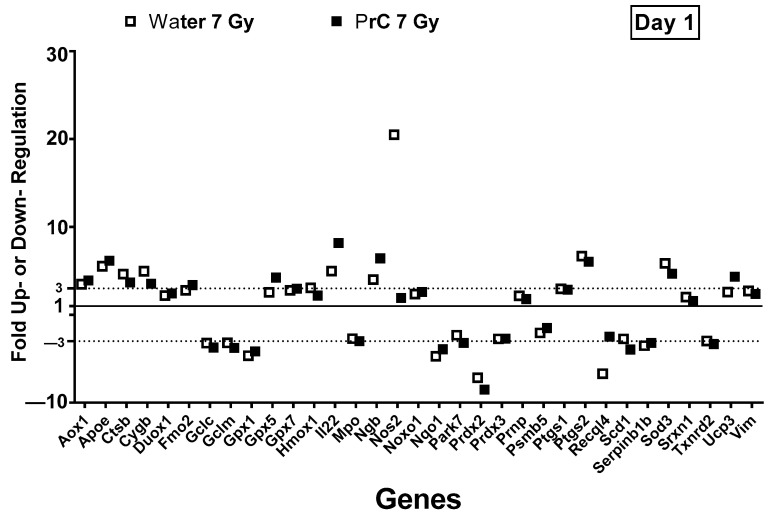
Differential expression levels of genes in the oxidative stress pathway and recovery by PrC-210 assessed by RTPCR. Serum samples from days 1, 7, and 14 post-TBI (7 Gy) were subjected to an array of 89 genes from the oxidative stress pathway. Dotted lines indicate threshold for significant difference. Gene expression analysis on day one showed a 20-fold up-regulation of Nitric oxide synthase 2 (Nos2) in irradiated water-treated mice. Irradiated mice administered PrC-210 significantly downregulated the Nos2 expression. This finding supports a Nos2 role in the radioprotective effects of PrC-210. Nos2 encodes an inducible enzyme that produces nitric oxide, a free radical and major messenger molecule.

PCR array-based expression levels of eighty-four ROS and antioxidant-regulated genes were investigated in mouse spleens affected by radiation-induced oxidative stress. Twenty genes were significantly upregulated, and fourteen were significantly downregulated (Figure 7) compared to naive control mice following irradiation. Genes are represented in alphabetical order with fold-change and p-value and a threshold of three-fold compared to non-irradiated controls in Supplementary Table S1. Antioxidant-related genes, Gpx5, Gpx7, Ctsb, Ptgs1, Ptgs2, Sod3, and Srxn1 were upregulated within 24 h post-radiation, however, Gpx1, Prdx2, Prdx3, Serpinb1b, and Txnrd2 were downregulated. PrC-210 pre-treatment did not show any differential regulation of these genes compared to irradiated controls, although they were all significantly different compared to naive control. Among the ROS-regulated genes, Sod3, Nos2, Noxo1, Aox1, Fmo2, IL22, Apoe, Duox1, Hmox1, Prnp, and Ucp3 were significantly upregulated compared to naive control day one following exposure, whereas Recql4, Scd1, Gclc, Gclm, Mpo, Nqo1, Park7, Prdx2, Psmb5 were significantly downregulated. Interestingly, Nos2 (inducible nitric oxide synthase 2) was highly induced (20-fold) following radiation, which was significantly attenuated by PrC-210 pre-treatment to the basal level. All other genes did not show differential response compared to irradiated control. Three oxygen-transporter genes (Cygb, Ngb, and Vim) were significantly upregulated following irradiation; they were not altered by PrC-210 treatment.

### 3.9. PrC-210 Pre-Treatment Restored Surviving Crypts, Reduced Bacterial DNA, Recovered Serum Citrulline and SAA Levels Following Radiation-Injury

**Figure 8:**
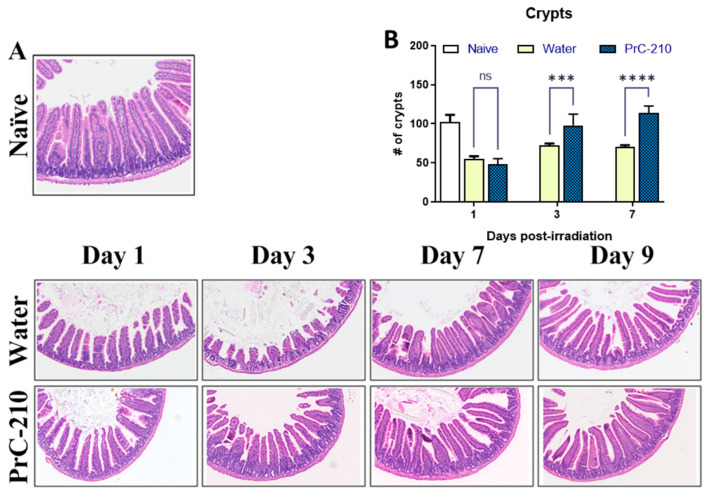
Histological evaluation of jejunum on days one, three, seven, and nine post-TBI. (**A**) H&E-stained representative sections of jejunum from naive, irradiated (9.75 Gy) water, and PrC-210-treated groups. Damage to villi due to radiation was observed by day three. (**B**) Viable crypts in all three groups were quantitated. Significant recovery in the PrC-210-treated group was observed when compared to the water-treated group on days three and seven by ANOVA (*** *p* ≤ 0.001, **** *p* ≤ 0.0001).

**Figure 9:**
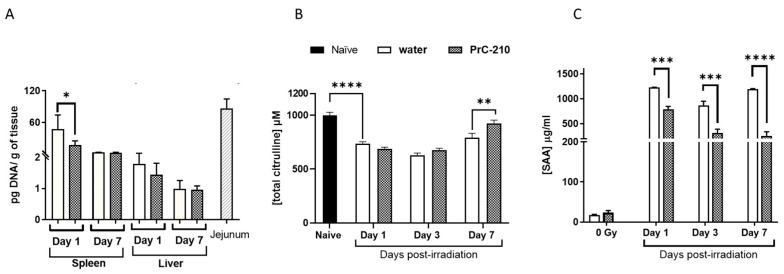
Protection of GI tissue by PrC-210. (**A**) Bacterial translocation of gut bacteria to liver and spleen assessed by PCR. Gut bacteria load in the spleen and liver were estimated in irradiated animals from both groups at both time points. There was a significant difference (*t*-test * *p =* 0.017) between the water-treated and PrC-210-treated groups on day one post-TBI in the spleen. (**B**) H&E-stained representative sections of jejunum from naïve. A significant increase (** *p* ≤ 0.01) in serum citrulline in the PrC-210 group compared to the control on day three. **(C)** PrC-210 administration inhibited the radiation-induced elevated synthesis of sepsis marker Serum Amyloid A (SAA) in mouse serum compared to water-treated animals. The levels of SAA were evaluated in serum from samples collected on days one, three, seven, and nine post-TBI by ELISA. Represented data are mean ± standard error of the mean (SEM) for n *=* 4 mice per group; *** *p* ≤ 0.001, **** *p* ≤ 0.0001. Plasma levels of SAA were very high following irradiation. The PrC-210 group showed significantly lower SAA levels compared to the water group on all three days tested.

Intestinal injury was assessed on days 1, 3, 7, and 9 following radiation exposure (Figure 8A,B). Crypt microcolony assay showed a statistically significant effect of pre-treatment with PrC-210 on the intestinal injury response on days three and seven (Figure 8B). Histopathological analysis of the stained jejunum sections shows less structural injury in the PrC-210 pre-treated mice compared to vehicle controls on all days (Figure 8A). Radiation exposure is associated with translocation of gut bacteria to both the spleen and the liver; however, this effect was significantly reduced in the spleens of PrC-210-treated animals compared to control mice on day one following radiation (Figure 9A). No difference in the bacterial load was observed between the treatment groups in the liver (Figure 9A). Serum citrulline (Figure 9B) and SAA (Figure 9C) levels were measured to assess the extent of intestinal injury following 9.75 Gy exposure. As expected, citrulline levels were reduced significantly following the PrC-210 treatment compared to naive controls after radiation, and on day seven, significantly higher levels of citrulline were observed in the PrC-210 pre-treated group compared to the water-treated group. Similarly, SAA levels were increased significantly starting from day one post-TBI in both irradiated groups compared to the naive control; however, SAA levels were significantly reduced on all three days (days one, three, and seven) in the PrC-210 pre-treated groups.

## 4. Discussion

The current study was undertaken to determine the safety and efficacy of PrC-210 as a prophylactic countermeasure, which is an unmet need for warfighters before sending them to harm’s way for rescue or cleanup operations in a radiological/nuclear-contaminated field. In a recent study, PrC-210 demonstrated efficacy in ICR mice as a prophylactic countermeasure, administered IP (intraperitoneal) 15 min before exposure, as well as a mitigator administered IP 24 h after radiation exposure [12]. The mitigating property of PrC-210 was also validated in a second mouse strain, C57BL/6, which also showed recovery from bone marrow and gastrointestinal injury [12]. Here, we confirm the efficacy of PrC-210 in CD2F1 mice administered as a single dose orally 60 min prior to a 70% lethal radiation exposure. First, we have shown that PrC-210 was safe at 2× higher doses than the dose used for efficacy studies. We have shown recovery of bone marrow progenitor cells, which are the most sensitive cells following radiation damage to the hematopoietic system. In addition, we have shown accelerated recovery of circulatory blood cells and sternal megakaryocytes with PrC-210 treatment. Our data show that the biomarkers of radiation-induced bone marrow damage (EPO and Flt3L) were ameliorated by oral pre-administration of PrC-210. It is well known that the hematopoietic system is extremely sensitive to ionizing radiation. In earlier studies, we showed that various classes of prophylactic agents (PLX-R18, CDX-301, GT3, BBT-059, Ex-RAD, JNJ-26366821) were able to rescue mice from lethality by suppressing gamma radiation-induced damage in the hematopoietic system [14,15,17,24,25]. In this report, our data indicate that oral, prophylactic administration of PrC-210 provided equal or better protection of mice from radiation-induced lethality by protecting their hematopoietic system.

Lethal radiation exposure causes loss of critical intestinal cell mass and is associated with decreased intestinal function leading to malabsorption, fluid and electrolyte imbalance, bacteremia, endotoxemia, and subsequent lethality [21,26,27]. Earlier reports show that Gamma-tocotrienol, a vitamin E analog, when administered 24 hr prior to irradiation, effectively protected intestinal cells from radiation-induced damage and apoptosis by preferential upregulation of anti-apoptotic genes and thereby helping maintain intestinal crypt cell integrity [21]. Here, we report that when animals (CD2F1 mice) were exposed to a 90% lethal dose of radiation, administration of PrC-210 60 min prior to exposure protected intestinal integrity by restoring the surviving jejunal crypt cells and villi structure. Bacterial translocation from the gut was protected by PrC-210 pre-treatment in the nearby spleen. In addition, radiation-induced GI and sepsis markers (Citrulline and SAA) were found to be reduced toward baseline levels by PrC-210 pre-treatment. These data suggest that PrC-210 pre-treatment affords a beneficial effect in protecting mice from radiation-induced GI injury.

Nitric oxide metabolism and its role in radiation response were reported earlier [28]. Reports in the literature showed that ionizing radiation causes a large increase in NO levels, and its excessive production can lead to cytotoxicity and induce widespread inflammation [29]. Upregulation of the inducible form of nitric oxide synthase (iNOS) or Nos2 was observed in cultured smooth muscle cells exposed to gamma radiation [30]. Ultraviolet ray exposure of skin cells was also found to induce Nos2 expression [31]. Furthermore, it was shown that increased NO production resulting from Nos2 gene transfection enhances radiation-induced apoptosis, and it is known that apoptosis occurs at early time points following exposure [32]. In this study, we showed that (i) Nos2 gene expression is highly induced in mouse spleen one day after radiation exposure, and (ii) Nos2 expression was significantly reduced by pre-treatment with PrC-210. This provides one of several explanations to explain PrC-210 protective efficacy.

## 5. Conclusions

In summary, the results of this study demonstrated that PrC-210 has multiple protective effects to significantly suppress radiation-induced hematopoietic and intestinal injury when administered pre-radiation. Our data indicate that the protective effects of PrC-210 may have been achieved by (i) accelerating hematopoietic regeneration, (ii) decreasing oxidative and nitrosative stress by enhancing the antioxidant defense regulated genes, (iii) protecting surviving jejunal crypts, and (iv) reducing circulatory inflammation markers. These findings indicate that PrC-210 is an effective prophylactic agent that should be developed using the FDA animal rule.

## Data Availability

All data generated or analyzed during this study are included in this published article and its supplementary information files.

## Supplementary Materials

The following are available online at https://www.mdpi.com/article/10.3390/antiox12071417/s1. Figure S1: Basic safety study with PrC-210. (A) Body weight of animals during study. There was no significant change in the body weight of the animals treated with PrC-210. (B) Peripheral blood cell counts compared between naive, water, and PrC-210 groups when administered with and without fasting on day 14. (C) Results of the serum levels of the renal and hepatic panels on day 14. For all the parameters tested, there were no significant differences between the naive group and the treated groups (water and PrC-210). Thus, PrC-210 at 800 mg/kg administered as PO was found to be safe. Represented data are mean ± standard error of the mean (SEM) for n = 5 mice. Table S1: Quantitative changes in oxidative stress genes in spleen post-TBI (7Gy).
