# Supplementary material for: PrC-210 Protects against Radiation-Induced Hematopoietic and Intestinal Injury in Mice and Reduces Oxidative Stress
Source: Antioxidants (Basel). 2023 Jul 13;12(7):1417. doi: 10.3390/antiox12071417 (PMC10376632; doi:10.3390/antiox12071417)
Supplement: Supplementary file 1 [file antioxidants-12-01417-s001.zip › antioxidants-2467074-supplementary.pdf]

Supplementary Figure S1:

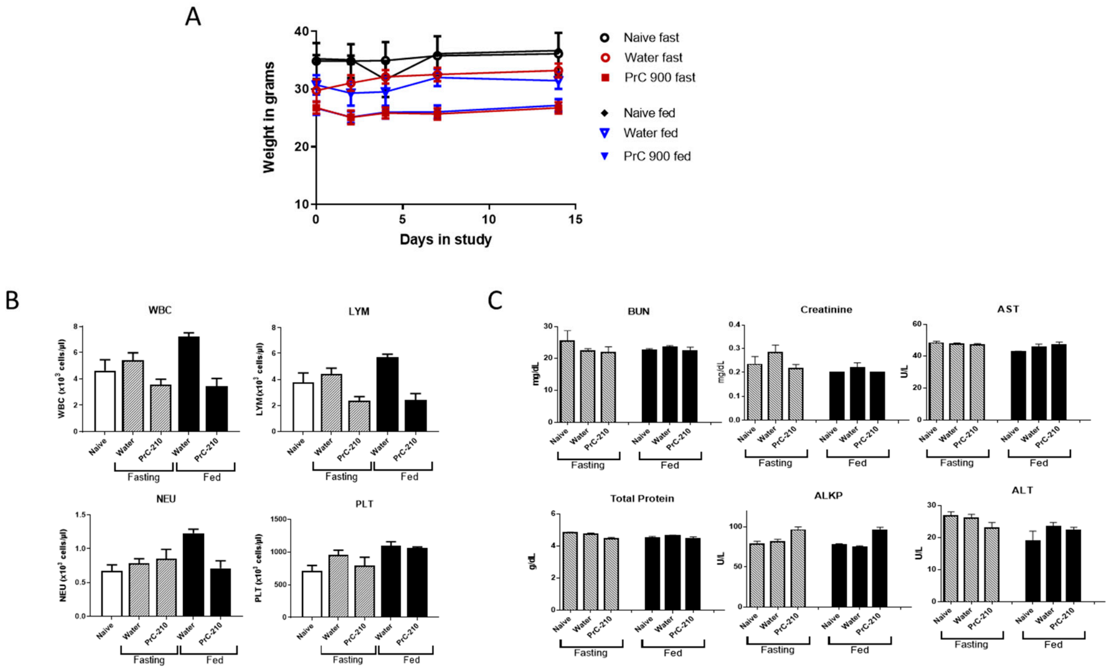

Supplemental Figure S1: Basic safety study with PrC-210. (**A**) Body weights of animals during study. There was no significant change in the body weights in the animals treated with PrC-210. (**B**) Peripheral blood cell counts compared between naïve, water and PrC-210 groups when administered with and without fasting on day 14. (**C**) Results of the serum levels of the renal and hepatic panel on day 14. In all the parameters tested there was no significant difference between the naïve group and the treated groups (water and PrC-210). Thus, PrC-210 at 800 mg/kg administered as PO was found to be safe. Data represented are mean ± standard error of the mean (SEM) for n = 5 mice.

Supplementary Table S1: Quantitative changes of oxidative stress genes in spleen post-TBI (7Gy)

Gene bank (Acc #)	Symbol	Gene description	Fold up- or down-regulation 7 Gy Water/ Naive	P value	Fold up- or down-regulation 7 Gy PrC-210/ Naive	P value
NM_009676	Aox1	Aldehyde oxidase 1	3.48	0.000191	3.9	0.000022
NM_009696	Apoe	Apolipoprotein E	5.53	0.000017	6.16	0.000005
NM_007798	Ctsb	Cathepsin B	4.63	0.000009	3.68	0.000033

NM_030206	Cygb	Cytoglobin	4.96	0.000022	3.55	0.000637
NM_001099297	Duox1	Dual oxidase 1	2.2	0.030954	2.44	0.048443
NM_018881	Fmo2	Flavin containing monooxygenase 2	2.78	0.027532	3.38	0.050603
NM_010295	Gclc	Glutamate-cysteine ligase, catalytic subunit	-3.22	0.000985	-3.72	0.000754
NM_008129	Gclm	Glutamate-cysteine ligase, modifier subunit	-3.19	0.000521	-3.77	0.000345
NM_008160	Gpx1	Glutathione peroxidase 1	-4.66	0.004636	-4.17	0.006207
NM_010343	Gpx5	Glutathione peroxidase 5	2.54	0.049167	4.25	0.203002
NM_024198	Gpx7	Glutathione peroxidase 7	2.78	0.000010	2.97	0.004323
NM_010442	Hmox1	Heme oxygenase (decycling) 1	3.07	0.000818	2.19	0.020642
NM_016971	Il22	Interleukin 22	4.97	0.038280	8.17	0.008504
NM_010824	Mpo	Myeloperoxidase	-2.72	0.010251	-3.01	0.020155
NM_022414	Ngb	Neuroglobin	4	0.008971	6.43	0.134694
NM_001313921	Nos2	Nitric oxide synthase 2, inducible	20.5	0.098704	1.93	0.195855
NM_172203	Noxo1	NADPH oxidase 1	2.37	0.001363	2.61	0.001790
NM_008706	Nqo1	NAD(P)H dehydrogenase, quinone 1	-4.72	0.000003	-3.92	0.000003
NM_020569	Park7	Parkinson disease (autosomal recessive, early onset) 7	-2.32	0.000549	-3.2	0.042703
NM_011563	Prdx2	Peroxiredoxin 2	-7.16	0.000066	-8.51	0.000058
NM_007452	Prdx3	Peroxiredoxin 3	-2.74	0.000729	-2.71	0.000600
NM_011170	Prnp	Prion protein	2.16	0.008296	1.8	0.039715
NM_011186	Psmb5	Proteasome (prosome, macropain) subunit, beta type 5	-2.06	0.003838	-1.5	0.047651
NM_008969	Ptgs1	Prostaglandin-endoperoxide synthase 1	2.96	0.000364	2.87	0.000948
NM_011198	Ptgs2	Prostaglandin-endoperoxide synthase 2	6.67	0.005442	6.04	0.000712
NM_058214	Recql4	RecQ protein-like 4	-6.71	0.000182	-2.48	0.305611
NM_009127	Scd1	Stearoyl-Coenzyme A desaturase 1	-2.75	0.003968	-3.95	0.003278
NM_173052	Serpinb1b	Serine (or cysteine) peptidase inhibitor, clade B, member 1b	-3.51	0.027676	-3.19	0.056307
NM_011435	Sod3	Superoxide dismutase 3, extracellular	5.85	0.000025	4.68	0.000189
NM_029688	Srxn1	Sulfiredoxin 1 homolog (S. cerevisiae)	2.03	0.000233	1.59	0.073245
NM_013711	Txnrd2	Thioredoxin reductase 2	-2.98	0.008525	-3.33	0.008514
NM_009464	Ucp3	Uncoupling protein 3 (mitochondrial, proton carrier)	2.59	0.000546	4.34	0.165068
NM_011701	Vim	Vimentin	2.72	0.000638	2.38	0.000712
